# Radiation damage in serial femtosecond crystallography studied in hemoglobin

**DOI:** 10.1107/S2059798326008569

**Published:** 2026-09-30

**Authors:** Marina Galchenkova, Ibrahim Dawod, Sebastian Cardoch, Janina Sprenger, Dominik Oberthür, Salah Awel, Emiliano De Santis, Oscar Grånäs, Juraj Knoska, Markus Metz, Spencer Passmore, Gisel Pena, Iosifina Sarrou, Max Wiedorn, Nicusor Timneanu, Henry Chapman, Oleksandr Yefanov, Carl Caleman

**Affiliations:** a Center for Free-Electron Laser Science (CFEL) Deutsches Elektronen-Synchrotron DESY Notkestrasse 85 22607Hamburg Germany; b Department of Physics and Astronomy, Uppsala University, Box 524, 75120Uppsala, Sweden; c European XFEL, Holzkoppel 4, 22869Schenefeld, Germany; d Department of Chemistry – BMC, Uppsala University, Box 576, 75123Uppsala, Sweden; e Department of Physics University of Rome Tor Vergata and INFN 00133Rome Italy; f Department of Physics and Astronomy, Swinburne University of Technology, Victoria3122, Australia; g Department of Physics, Universität Hamburg, 22761Hamburg, Germany; h Centre for Ultrafast Imaging, Universität Hamburg, 22761Hamburg, Germany; University of Oxford, United Kingdom

**Keywords:** X-ray free-electron lasers, serial femtosecond crystallography, radiation damage, X-ray pulse duration, global and specific damage

## Abstract

A central question in macromolecular crystallography is what X-ray free-electron laser pulse duration is required to obtain *damage-free* structural information. We compare serial femtosecond crystallography data from hemoglobin crystals using 3 and 10 fs pulses at comparable peak intensities (∼3 × 10^17^ W cm^−2^) and fixed photon energy (7.15 keV). Structural refinement produced very similar models and resolution-dependent data-quality indicators remained comparable. Both datasets also retained sufficient anomalous signal for phasing. These observations support the conclusion that high-resolution scattering was preserved under both pulse durations. Hybrid collisional–radiative and molecular-dynamics simulations show that under typical experimental conditions, atomic form-factor changes are negligible (<1%) and atomic displacements fall below the resolution limit imposed by the X-ray photon energy and the geometry of the detector. The combined experimental and theoretical results indicate that 10 fs pulses are adequate to obtain damage-free protein structures using femtosecond crystallography under the intensity conditions explored.

## Abstract 2

Protein structures determined by serial femtosecond crystallography are indistinguishable when measured with 3 and 10 fs X-ray pulses, indicating no structural benefit from further shortening the pulse duration under the studied conditions.

## 1. Introduction

X-ray radiation damage to structures has been a central topic of macromolecular crystallography (Holton, 2009 ▸; Garman & Weik, 2023 ▸; Shelley & Garman, 2024 ▸), as this process limits resolution and introduces changes that can mislead interpretation (Taube *et al.*, 2019 ▸; Pfanzagl *et al.*, 2020 ▸). In recent decades, X-ray free-electron lasers (XFELs) have been used to obtain structures by exposing crystals to radiation doses orders of magnitude higher than the levels traditionally considered acceptable for room-temperature data collection (Henkel & Oberthür, 2024 ▸). This approach, known as serial femtosecond crystallography (SFX), involves delivering a continuous flow of individual crystals and exposing them in random orientations to single X-ray pulses with typical intensities of 10^9^–10^11^ photons µm^−2^ (corresponding to 10^2^–10^4^ J cm^−2^ at a photon energy of 7.15 keV) that last from several tens to just a few femtoseconds (Chapman *et al.*, 2011 ▸; Boutet *et al.*, 2012 ▸). During the interaction, elastically scattered X-rays create a diffraction pattern that accumulates into Bragg peaks as long as atomic positions remain correlated. Because of the short pulse duration, the structures are nearly radiation damage-free (Williams *et al.*, 2025 ▸), enabling the investigation of very small crystals (Gati *et al.*, 2017 ▸; Williamson *et al.*, 2023 ▸) and of time-dependent phenomena (Kupitz *et al.*, 2014 ▸; Tenboer *et al.*, 2014 ▸; Brändén & Neutze, 2021 ▸; Christou *et al.*, 2023 ▸).

Structures are not necessarily completely free of radiation damage because at the photon energies commonly used in macromolecular crystallography of about 10–20 keV, photoabsorption is roughly one order of magnitude more probable than elastic scattering (Doniach, 1996 ▸; Neutze *et al.*, 2000 ▸; Chapman *et al.*, 2014 ▸). A single photoionization event can initiate a cascade of secondary electron-impact ionization that causes further electronic damage via the creation of highly excited configurations and structural damage from bond breakage and increased Coulomb forces, transforming the sample into plasma within the pulse (Caleman *et al.*, 2011 ▸; Chapman *et al.*, 2006 ▸). Direct (on molecule) and indirect (on solvent) damage events lead to global changes in the structure (such as unit-cell expansion, reduction in Bragg peak photon counts, increase in *B* factors) as well as specific changes (such as metal-ion reduction, disulfide-bond breakage) (Garman & Weik, 2023 ▸). In SFX, global damage breaks spatial coherence and terminates the Bragg signal in a process known as *diffraction before destruction* (Barty *et al.*, 2012 ▸; Caleman *et al.*, 2015 ▸). The extent of radiation damage present in SFX experiments is still not fully understood. Specific radiation damage might not always be possible to avoid, as experimental and theoretical studies have indicated (Lomb *et al.*, 2011 ▸; Nass *et al.*, 2015 ▸, 2017 ▸, 2020 ▸; Ebrahim *et al.*, 2019 ▸; Caleman *et al.*, 2020 ▸; Williams *et al.*, 2025 ▸). For the present study the most related study is that of Williams and coworkers, which shows that although some site-specific damage signals can be detected, they do not significantly affect the overall protein structure or key bond lengths under typical experimental conditions, supporting the diffraction before destruction assumption. An important experimental observation for SFX radiation damage is presented by Nass and coworkers, where they describe how sulfurs in disulfide bonds might undergo correlated motion, leading to diffracted signal even if the atoms have actually moved away from their native position (Nass *et al.*, 2020 ▸). This observation indicates the need for short X-ray pulses in SFX, since coherent atom dynamics in the crystal might give rise to ‘false’ Bragg signal. X-ray pulse durations shorter than a few femtoseconds, comparable to the Auger decay lifetime, might be needed to outrun radiation damage caused by secondary ionization (Son *et al.*, 2011 ▸). On the other hand, compressing the same number of photons into shorter pulses, that is increasing the intensity, can lead to transparency due to inner shell vacancy formation and other nonlinear phenomena (Young *et al.*, 2010 ▸; Hoener *et al.*, 2010 ▸; Nass, 2019 ▸). The total diffraction signal depends not only on the photon count but also on the size of the crystal. When the beam is focused to a spot smaller than the crystal, the signal scales in proportion to the crystal thickness, and measurements with smaller crystals generally require pulses with more photons. Estimating the optimal pulse duration to use, taking into account the dependence of nonlinearities and structural destruction on pulse intensity, is therefore a nontrivial exercise.

**Figure 1:**
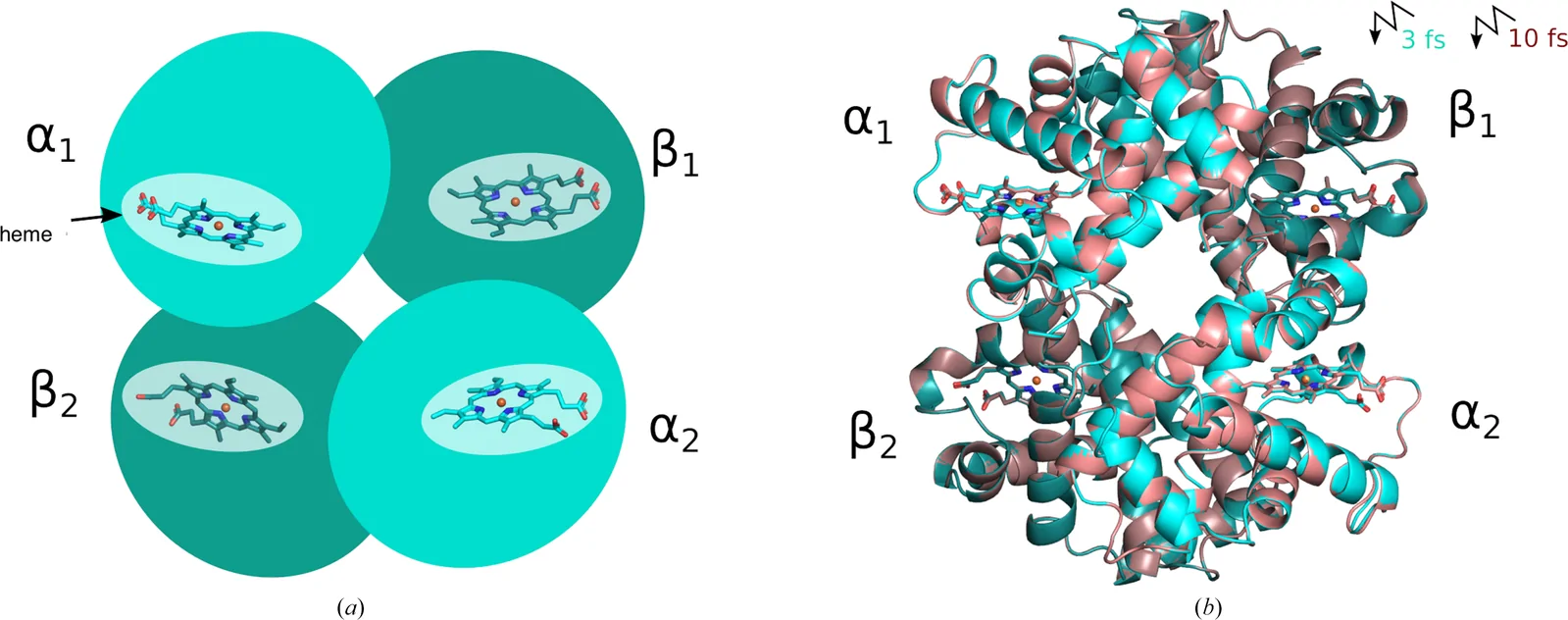
(*a*) Schematic structure of tetrameric (α_2_β_2_) hemoglobin highlighting the positions of the heme groups. (*b*) Overlay of structures of horse hemoglobin refined against data collected with 3 fs (cyan cartoon) and 10 fs (brown cartoon) XFEL pulse durations. The β-subunits are in darker tones compared with the corresponding α-subunits. The heme groups are represented as sticks with carbon in the respective color of the backbone (brown or cyan), oxygens in red, nitrogens in blue and the central iron as an orange sphere.

This study aimed to evaluate how radiation-induced damage affects the resolution of protein structures reconstructed from SFX measurements of horse hemoglobin crystals. Fig. 1 ▸(*a*) shows an illustration of the tetrameric hemoglobin structure. Each of the four subunits (two α-subunits and two β-subunits) contains heme groups consisting of a porphyrin ring with a central Fe^2+^ ion. Measurements were conducted with pulse durations of 3 and 10 fs of similar intensities. In addition, we measured at higher fluence with the 10 fs pulse duration. The objective of this experiment was to evaluate the influence of pulse duration on the reconstruction and identify potential radiation damage around the iron centers. For this reason, we only focus on the 3 fs and attenuated 10 fs datasets, which have comparable intensities of ∼3 × 10^17^ W cm^−2^. In addition, the photon energy was tuned to 7.15 keV, just above the Fe *K*-shell absorption edge. Fig. 1 ▸(*b*) demonstrates an overlay of the two experimental structures reconstructed from the measurements. We also performed collisional–radiative (Scott & Mayle, 1994 ▸; Scott, 2001 ▸) and molecular-dynamics simulations (Dawod *et al.*, 2024 ▸) to evaluate global and specific damage caused by the two different pulse durations. We examined pulse-weighted ionization, atomic scattering factors and root-mean-squared displacement, exploring fluence values beyond those used in our experiments. Pulse-weighted quantities were obtained by averaging the instantaneous values over time, weighted by the Gaussian temporal envelope of the pulse. This approach highlighted changes that occur during the pulse, particularly during periods of higher intensity.

## 2. Experimental methods

### 2.1. Crystal preparation

Equine hemoglobin (Sigma, CAS-9047090) stock solution at 8–10 mg ml^−1^ was prepared in 50 m*M* HEPES pH 7.5 and precipitated using the stirred-batch method (Tenboer *et al.*, 2014 ▸) by mixing into 24–26%(*w*/*v*) PEG 3350 in a 1:1 ratio. The solution was kept at room temperature while it was continuously stirred. Crystals appearing in about 2 h were filtered using 2 µm stainless-steel filters (Upchurch) and were quenched with 25%(*w*/*v*) PEG 3350 for immediate use, as shown in Supplementary Fig. S1. While hemoglobin usually crystallizes in a *C*-centered monoclinic form, the crystals used here exhibited orthorhombic symmetry (*P*2_1_2_1_2_1_).

### 2.2. Data collection and processing

Experimental data for hemoglobin samples from different batches were collected at the LCLS MFX experimental station at the SLAC National Accelerator Laboratory in Menlo Park, California, USA (Boutet *et al.*, 2016 ▸) during beamtime LR17 (Knoška *et al.*, 2020 ▸; Trost *et al.*, 2023 ▸). The experiments were conducted using a repetition rate of 120 Hz and a beam focus size of roughly 4 µm. The pulse energy, accounting for beamline transmission losses, was estimated to be 0.1 and 1.5 mJ for the 3 and 10 fs pulses, respectively (Knoška *et al.*, 2020 ▸; Trost *et al.*, 2023 ▸). The crystal solution was streamed across the X-ray beam focus as a liquid jet formed using a DFFN nozzle (Oberthuer *et al.*, 2017 ▸) on the *RoadRunner* system, specifically adapted for liquid jets (Roedig *et al.*, 2017 ▸; Knoška *et al.*, 2020 ▸). To minimize the background scattering, a capillary beamstop and a helium enclosure were employed (Meents *et al.*, 2017 ▸).

The diffraction was collected using the Cornell–SLAC pixel-array detector (CS-PAD; Hart *et al.*, 2012 ▸). A typical diffraction pattern is shown in Supplementary Fig. S2. The detector geometry was refined to pixel precision using *geoptimiser* to enhance the quality of data analysis (Yefanov *et al.*, 2015 ▸). Real-time monitoring was performed using the *OnDA* tool to assess the hit fraction and data quality on the fly (Mariani *et al.*, 2016 ▸). The collected diffraction patterns, identified as *hits* (containing crystal diffraction), were saved to HDF5 files using the *Cheetah* software (Barty *et al.*, 2014 ▸) for further processing.

**Figure 2:**
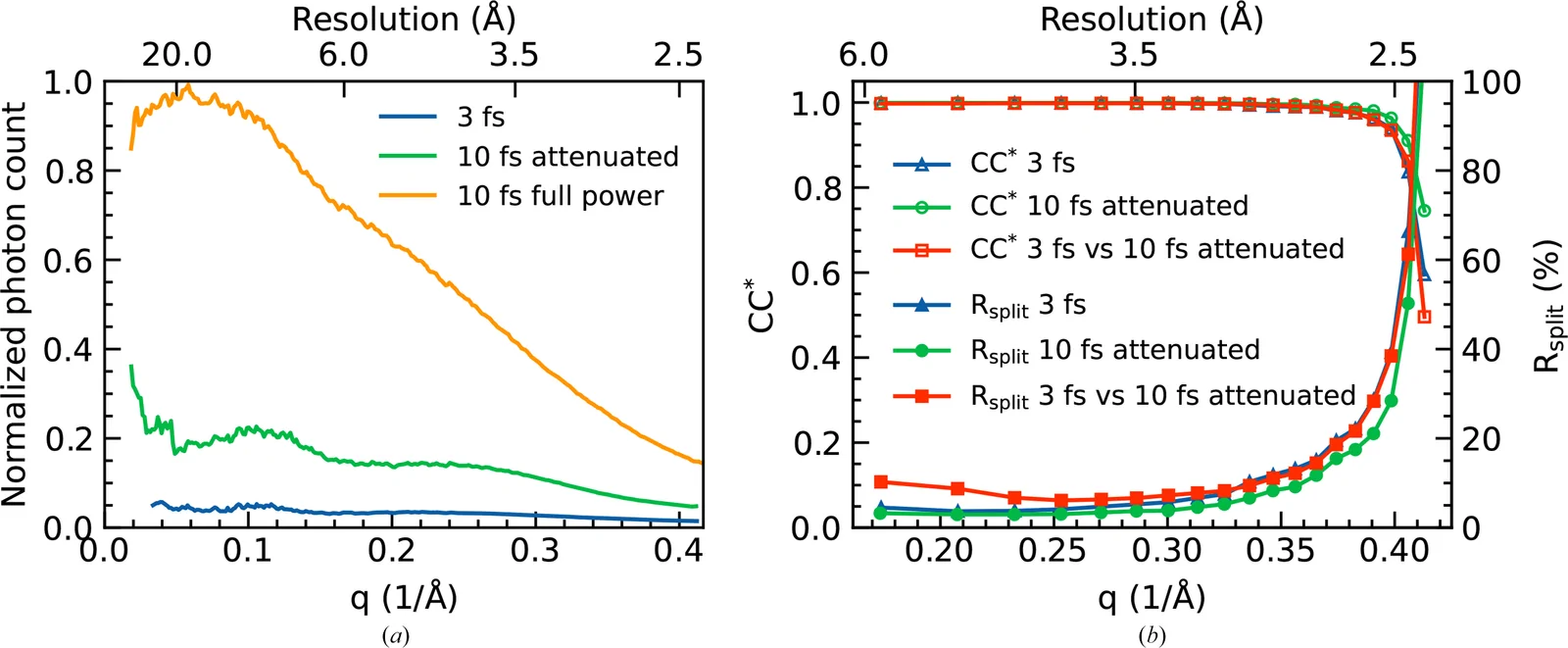
(*a*) The normalized average photon counts of the Bragg peaks in three measured datasets: 3 fs pulses (2.7 × 10^17^ W cm^−2^), 10 fs attenuated pulses (3.4 × 10^17^ W cm^−2^) and 10 fs full power pulses (1.3 × 10^18^ W cm^−2^). (*b*) The data-quality metrics CC* and *R*_split_ for the datasets measured with 3 fs pulses, 10 fs attenuated pulses and between the halves of the two datasets.

The 3 fs pulses were generated using electron bunches of lower charge than for the 10 fs pulses. This resulted in a corresponding decrease in pulse energy. To compensate for the drop in the number of photons per pulse, the measurements with 10 fs pulses were carried out in two modes: attenuated and full power. The 10 fs data were then sorted according to the incident intensity and analyzed separately. The average photon counts of the Bragg peaks, with respect to the 3 fs dataset, differ by factors of 4.3 (attenuated) and 17.4 (full power), as can be seen from Fig. 2 ▸(*a*).

For the structure comparison, we focused on a subset of samples from the same batch (HF2) that experienced the same crystallization conditions. These samples exhibited similar statistics regarding the number of indexed crystals for both pulse durations, as shown in Table 1 ▸. The full histograms of the photon counts of the found peaks (peakograms) are presented in Supplementary Fig. S3.

The data were processed with *CrystFEL* (White *et al.*, 2012 ▸), using the *indexamajig* program (version 0.9.1+0e48c77b). The *peakfinder*8 algorithm was used to detect Bragg peaks with the following parameters: --min-snr=6, --threshold=200,--min-pix-count=1. The identified hits were indexed using *XGANDALF* (Gevorkov *et al.*, 2019 ▸) with the --multi and --no-check-peaks options. An illustration of the unit-cell distributions for selected subsets with 3 and 10 fs pulse duration can be found in Supplementary Fig. S4.

To scale and merge the data into the point group *mmm* (and 222 for analysis of the anomalous signal), we utilized the *partialator* program (version 0.9.1+0e48c77b) of *CrystFEL*. Three iterations were performed with the parameters --push-res=1.0 and --model=xsphere. The quality assessment of the data involved the calculation of figures of merit using *CrystFEL*’s *compare_hkl* (including *R*_split_, CC_1/2_ and CC*) and *check_hkl* (including signal-to-noise ratio, multiplicity and completeness) as shown in Fig. 2 ▸(*b*). MTZ files for crystallographic data processing were generated from the *CrystFELhkl* files using *F*2*MTZ* from *CCP*4 (Winn *et al.*, 2011 ▸).

**Figure 3:**
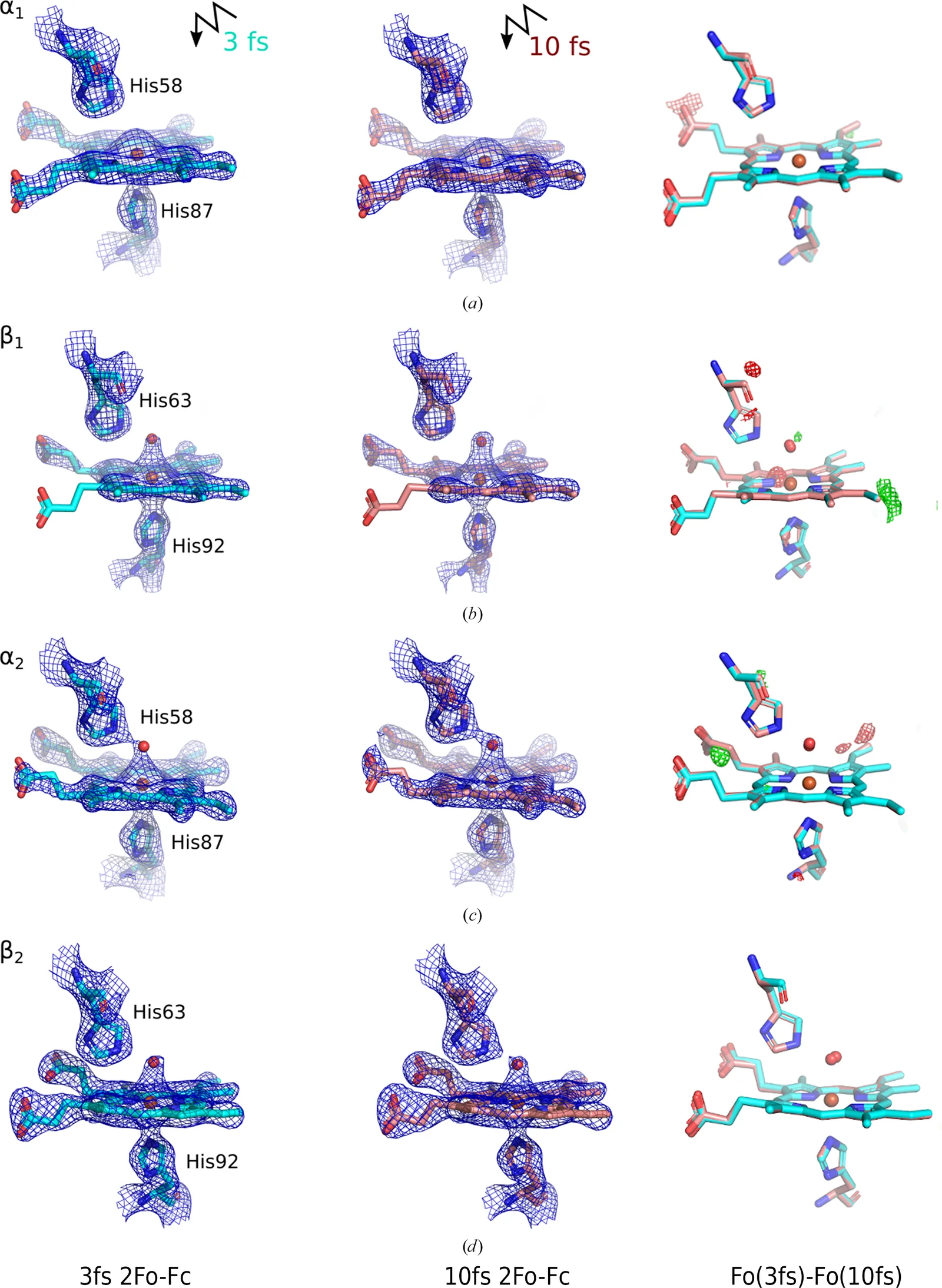
Stick models of the heme group with coordinating histidine residues from all subunits of horse hemoglobin after 3 and 10 fs as an example. The left (3 fs) and central (10 fs) panels show the 2*F*_o_ − *F*_c_ electron-density maps at 1.5σ, carve = 1.6. The right panel shows the overlaid structures with *F*_o_(3 fs) − *F*_o_(10 fs) electron-density maps at 3.0σ, carve = 1.8.

Molecular replacement was performed using *Phaser* (McCoy *et al.*, 2007 ▸) with PDB entry 6r2o as the search model. The resulting structures were iteratively refined using *phenix.refine* (Adams *et al.*, 2010 ▸) and *Coot* (Emsley *et al.*, 2010 ▸). The 2*F*_o_ − *F*_c_ electron-density maps for chains *A*–*D* of hemoglobin are presented in Fig. 3 ▸. Experimental phasing was carried out using *SHELXC*/*D*/*E* (Sheldrick, 2002 ▸, 2008 ▸, 2010 ▸; Schneider & Sheldrick, 2002 ▸), density modification, refinement with *REFMAC* (Murshudov *et al.*, 1997 ▸) and model building with *Buccaneer* (Cowtan, 2006 ▸) in the *CRANK*2 pipeline from the *CCP*4 suite (Agirre *et al.*, 2023 ▸; Potterton *et al.*, 2018 ▸).

## 3. Computational methods

### 3.1. Estimating the crystal density

To simulate a realistic crystal structure, we first determined the crystal’s hydration and density using the molecular-dynamics package *NAMD* (Phillips *et al.*, 2020 ▸) together with the CHARMM36 force field (Best *et al.*, 2012 ▸) and TIP3P water model. We solvated the crystal structures with different numbers of water molecules. We performed an equilibration procedure starting with energy minimization, followed by 200 ps constant volume and temperature simulations at 298 K, and completed by 200 ps simulations with constant temperature and pressure using Langevin piston Nose–Hoover pressure coupling at 0 bar with a flexible cell in all directions. The results shown in Supplementary Fig. S5 revealed that we could preserve the original crystallographic volume with 7986 water molecules corresponding to a mass density of ∼1.18 g cm^−3^. We used the resultant stoichiometry and density as input in collisional–radiative simulations, and the atomic positions as input for radiation-damage molecular-dynamics simulations.

### 3.2. Collisional–radiative simulations

We carried out collisional–radiative simulations (Scott & Mayle, 1994 ▸; Scott, 2001 ▸) to evolve electronic populations and free-electron densities over time. The code has been previously used to simulate different XFEL experiments on crystal structures (Barty *et al.*, 2012 ▸; Beyerlein *et al.*, 2018 ▸; Makita *et al.*, 2019 ▸; Cardoch & Timneanu, 2025 ▸). The simulations consider the existing material properties and the incoming X-ray pulse to iteratively reach a self-consistent solution. To simplify the calculations, we described the X-rays as a Gaussian temporal profile with a pulse duration defined by its full-width at half-maximum and a flat-top bandwidth profile Δ*E*/*E* = 1 × 10^−3^.

The simulations model the protein crystal as a one-dimensional 4 µm continuum with a density and stoichiometric composition defined by the molecular-dynamics procedure outlined in Section 3.1. Each element was modeled using screened hydrogenic atomic data with a principal quantum number *n* and orbital angular momentum quantum number *l* level description (Scott & Hansen, 2010 ▸). The model incorporates core and valence multiply excited configurations, as well as photoionization, photoexcitation, autoionization, electron-impact excitation and electron-impact ionization. Photoionization and photoexcitation cross sections are determined from oscillator strengths (Scott & Hansen, 2010 ▸; Chung *et al.*, 2016 ▸). Electron collisional excitations and ionizations come from fits to *JJATOM* (Chung *et al.*, 2007 ▸) and Sampson & Golden (1971 ▸), respectively. Treatment for autoionizing states follows from *FLYCHK* (Chung *et al.*, 2005 ▸).

The free electrons follow a Maxwellian energy distribution, which assumes that photoelectrons instantly thermalize. This approximation fails to capture photoelectron relaxation and tends to overestimate electron-impact ionization within the first few femtoseconds (Cardoch & Timneanu, 2025 ▸). For this reason, we omitted secondary ionization for iron. Other investigations on protein crystals exposed to XFELs have shown that within femtoseconds, heavy atoms are primarily photoionized and electron collisions do not severely affect their ionization (Passmore *et al.*, 2025 ▸). The lack of collisionally ejected electrons originating from these heavy atoms also does not influence the ionization of neighboring light atoms (Passmore *et al.*, 2025 ▸).

### 3.3. Atomic scattering factors

We used density-functional theory (Wills *et al.*, 2010 ▸) with the PBE96 (Perdew *et al.*, 1996 ▸) exchange–correlation functional to compute isolated-atom spherically symmetric electron densities for a given electronic configuration. We performed this calculation for all electronic configurations included in the collisional–radiative simulations. The electronic structure calculations needed magnetic quantum number information, while the *n*, *l* hydrogenic atomic data lacked this information. We solved this discrepancy by first populating states with the lowest total angular momentum. From the electron density, we calculated atomic scattering factors based on equation (16) from Son *et al.* (2011 ▸).

### 3.4. Radiation damage-resolved molecular-dynamics simulations

We used *MolDStruct* (Dawod *et al.*, 2024 ▸), a simulation tool built on the classical molecular-dynamics software *GROMACS* version 4.5.4 (Van Der Spoel *et al.*, 2005 ▸), which is capable of resolving radiation damage in protein crystals. The code couples ion populations and free-electron temperature and density from collisional–radiative simulations with molecular dynamics. We used the CHARMM36 force-field (Vanommeslaeghe *et al.*, 2010 ▸) parameters to model bonded and nonbonded interactions. Electrons are not treated explicitly. Instead, we incorporate the plasma environment by dynamically updating atomic charges and the potential energy landscape based on the ion population, free-electron temperature and density. For nonbonded interactions, we screen the Coulomb and Lennard–Jones potentials using a hybrid screening that, based on plasma conditions, switches between an ion-sphere model for strongly coupled plasmas and a Debye model for weakly coupled plasmas. For bonded interactions, we retain the Coulomb potential, omit the Lennard–Jones potential and replace the harmonic potential with a Morse potential that approaches zero at large separation distances. We model the weakening of covalent bonds by scaling the potential by a factor 1 − *z*, where *z* is the degree of ionization between the bonded atoms, going from 0 (neutral) to 1 (fully ionized). Our approach has been benchmarked to experiments (Beyerlein *et al.*, 2018 ▸; Nass, 2019 ▸; Dawod *et al.*, 2024 ▸). Similar methods have been used to investigate the displacement of atoms in protein crystals exposed to intense femtosecond X-rays from XFELs (Jurek *et al.*, 2016 ▸; Kozlov *et al.*, 2020 ▸; Nass *et al.*, 2020 ▸).

## 4. Results

### 4.1. Comparison between 3 and 10 fs measurements

Comparison of the processed data sets obtained from the two pulse durations (3 and 10 fs with ∼3 × 10^17^ W cm^−2^) allows an initial estimation of their similarity. The processing results are summarized in Table 1 ▸ (an extended version of the table can be found as Supplementary Table S1), and are visualized in Fig. 2 ▸(*b*) and Supplementary Fig. S3. The data-collection and processing statistics do not show significant differences and the unit-cell parameters are very similar. The resolution of the reconstructed structures (2.46 Å) was limited by the energy of X-rays (7.15 keV) and the geometry of the experiment for both data sets (see Supplementary Table S1 for the data statistics). The data metrics *R*_split_ and CC*, calculated between two different datasets measured with two pulse durations (3 fs versus 10 fs; Fig. 2 ▸*b*), demonstrate good correlation of the measured intensities of the structure factors of the two datasets, except for the region at low resolution. Also, the graphs of *R*_split_ and CC* between the two different datasets are not worse than the consistency graph of the same metrics for the two individual datasets (Fig. 2 ▸*b*), which further suggests a high similarity between the two measurements. The refinement of the two structures results in very similar structures. For refinement, an identical resolution range of 2.46–29.16 Å was used and the refinement statistics (Table 1 ▸) do not show significant differences, which allows us to compare the two structures.

To inspect whether any minor signs of radiation damage are present, we evaluated the electron density around the heme groups and the coordinating His residues. Fig. 3 ▸ shows the respective 2*F*_o_ − *F*_c_ density maps around subunit α_1_ as well as the difference densities *F*_o_ − *F*_o_ between the 3 and 10 fs data sets. The two 2*F*_o_ − *F*_c_ maps and their structural models look very similar for both data sets. Also, in the difference maps there are no signs of significant changes (see the overall unbiased *F*_o_ − *F*_o_ map, calculated with phases derived from experimental phasing, in Supplementary Fig. S6), and the heme-group structures align perfectly. The same applies to the electron densities of all heme groups in the structures in Fig. 3 ▸.

### 4.2. The anomalous signal for both 3 and 10 fs datasets allows phasing

Both datasets could be phased with the *SHELXC*/*D*/*E* (Sheldrick, 2002 ▸, 2008 ▸, 2010 ▸; Schneider & Sheldrick, 2002 ▸) implementation in *CRANK*2 from *CCP*4 (Agirre *et al.*, 2023 ▸; Potterton *et al.*, 2018 ▸), using the single-wavelength anomalous diffraction (SAD) signal of the Fe atoms. The photon energy used was just above the iron absorption edge. The calculated CC_ano_ was 0.187 for the data collected with 10 fs pulses and 0.141 for the 3 fs pulses (a resolution-dependent plot of CC_ano_ can be found in Supplementary Fig. S7). Even though the strength of the anomalous signal was quite low (especially at low and high resolution), it was still sufficient for automatic model building and refinement in *CRANK2* [using *Buccaneer* (Cowtan, 2006 ▸) and *REFMAC* (Murshudov *et al.*, 1997 ▸) respectively]. It took significantly more rounds of iterative refinement/model building after initial chain tracing with *SHELXE* (Sheldrick, 2002 ▸) for the *R* factor of the 3 fs dataset to drop below 0.45 than for the 10 fs dataset (298 rounds versus 21 rounds; see Supplementary Fig. S8). This can be attributed to weaker initial phases (see also subtle differences in anomalous peak height, see Supplementary Table S2, that are not clearly visible in the anomalous difference electron- density maps, see Supplementary Figs. S9 and S10). However, using the same parameters in *CRANK*2, the final *R* factor is slightly lower for the 3 fs dataset (0.215 versus 0.229).

### 4.3. Theoretical electronic damage comparison between 3 and 10 fs

**Figure 4:**
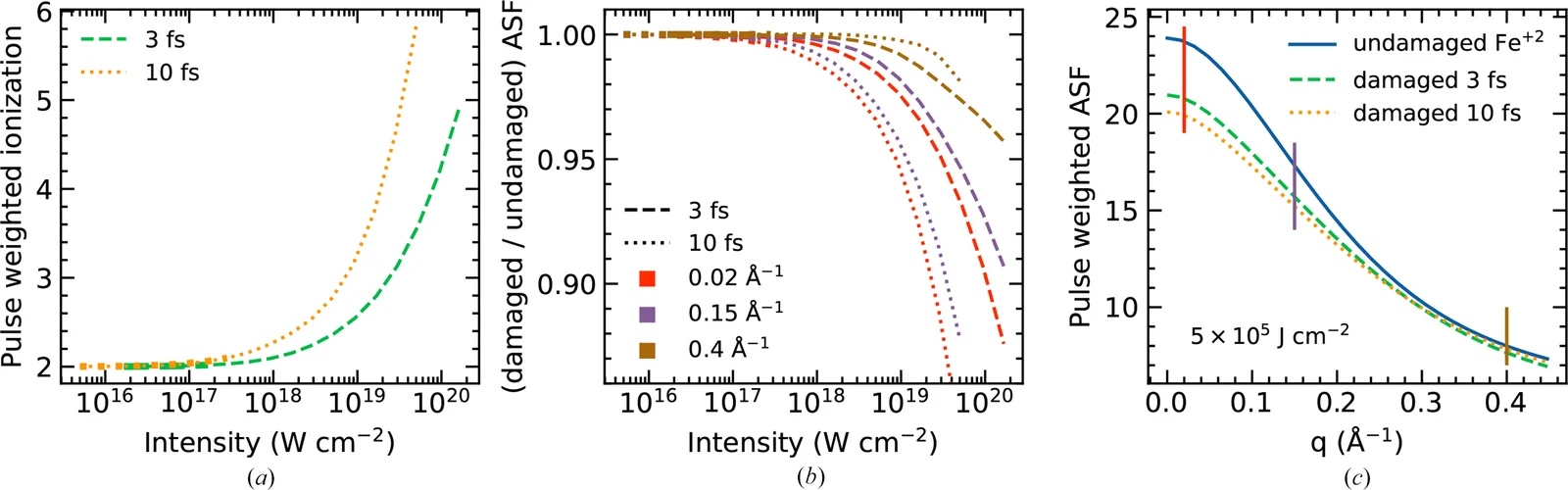
Comparing iron’s pulse-weighted ionization and atomic scattering factor (ASF) for 3 fs (dashed) and 10 fs (dotted) pulse durations. Thicker lines correspond to intensities accessed by the experiment. (*a*) Ionization as a function of different incident X-ray intensity. (*b*) Ratio of *damaged* over *undamaged* Fe^2+^ ASF at three selected *q* = 0.02 Å^−1^ (red), *q* = 0.15 Å^−1^ (purple) and *q* = 0.40 Å^−1^ (brown) crystallographic reflections. (*c*) Damaged and undamaged ASF at the highest simulated fluence of 5 × 10^5^ J cm^−2^, corresponding to 1.7 × 10^20^ W cm^−2^ for a 3 fs pulse and 5.0 × 10^19^ W cm^−2^ for a 10 fs pulse.

We assessed electronic damage via the ionization dynamics and changes to the atomic scattering factor during the X-ray pulse. We explored an intensity range with a lower limit of 5 × 10^15^ W cm^−2^ for the 10 fs pulse and 1 × 10^16^ W cm^−2^ for the 3 fs pulse, extending to upper limits well beyond the experimental conditions. Across this range, simulations for both pulse durations predicted minimal electronic damage at pulse intensities around 3 × 10^17^ W cm^−2^ and below. Fig. 4 ▸(*a*) illustrates that iron’s ionization averaged over time by the X-ray pulse shape (pulse-weighted) remained effectively unchanged from its initial Fe^2+^ charge state. Here, we plot the quantities related to atomic scattering factors in terms of the pulse intensity, which indicate the onset of disruption of the electronic structure of atoms but do not yet show how the diffraction pattern of the crystal will change due to atomic displacements. Simulations corroborated experimental findings and showed no difference in electronic damage caused by the two pulse durations around the heme group. This is also the case for other atomic species, shown in Supplementary Fig. S11, where their average ionization stayed below +0.1. Iron’s ionization evolved similarly for both pulse durations. To further track the onset of ionization, we plot the atomic structure factor normalized by its value for the ground-state Fe^2+^ atom in Fig. 4 ▸(*b*) for three *q*-values. It is seen that these remain close to unity for intensities less than 1 × 10^18^ W cm^−2^. The term *damaged* refers to the atomic structure factor weighted by both the electronic occupation distribution computed from collisional–radiative simulations and the Gaussian X-ray pulse profile. The term *undamaged* refers to the Fe^2+^ ground-state atomic structure factor.

Simulations indicated that damage to the metal center would begin taking place above 1 × 10^18^ W cm^−2^, where low-*q* reflections were most severely affected, yet differences between pulse durations remained minimal. In general, we found that higher *q*-values were more tolerant to radiation damage. This behavior was also theoretically predicted for silicon crystals (Cardoch & Timneanu, 2025 ▸). At a threshold of 1 × 10^19^ W cm^−2^, when the charge state reached Fe^3+^, clear differences between the two pulse durations emerged. For high-*q* reflections the 3 fs pulses were most detrimental. Conversely, for low-*q* reflections the 10 fs pulses were most detrimental. Fig. 4 ▸(*c*) shows the predicted differences in atomic scattering factors for iron for the two pulse durations and the highest theoretical fluence explored. These changes can be attributed to the interplay between photoexcitation and radiative and nonradiative relaxation. Based on values from the atomic model, after the creation of a 1*s* vacancy in iron, the Auger–Meitner decays proceed mainly via *K*–*L*_1_*L*_2,3_ in 1.47 fs and radiatively with a yield of 0.340 (Krause, 1979 ▸) mainly via *K*–*L*_3_ in 1.85 fs. The short pulse, therefore, interacts with a structure in a more core-excited state, leading to greater changes in the atomic structure factor at high *q*. In contrast, the long pulse interacts with iron in a less core-excited but more ionized configuration since the atoms have had time to relax and be photoexcited multiple times within the pulse duration.

### 4.4. Theoretical structural damage comparison between 3 and 10 fs

**Figure 5:**
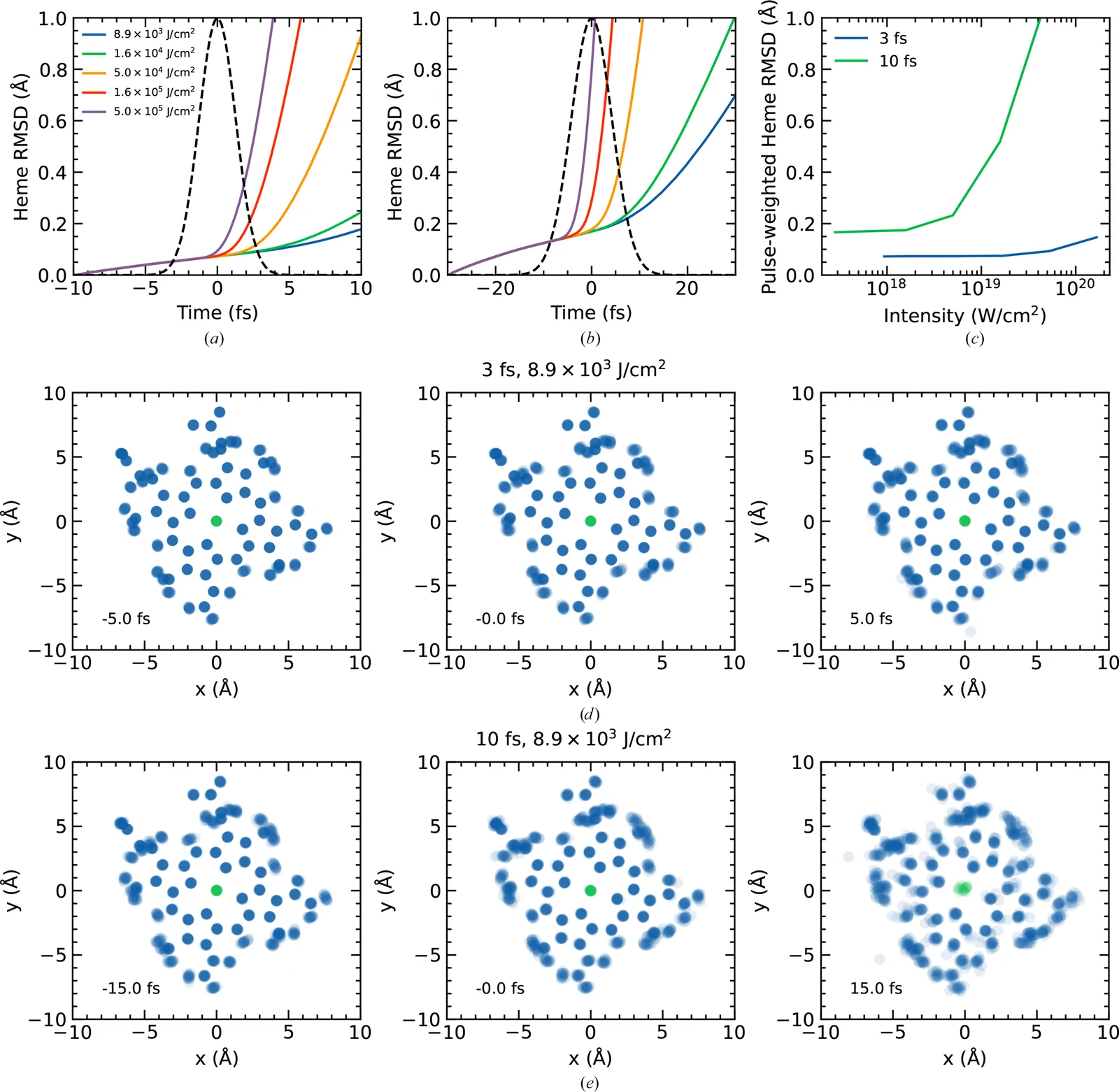
Root-mean-square displacements (r.m.s.d.s) of the heme group at different fluence values with (*a*) 3 fs and (*b*) 10 fs assumed pulse duration. The dashed lines outline the assumed pulse profile. The r.m.s.d.s correspond to an average over multiple trajectories with different starting velocities. (*c*) Pulse-weighted r.m.s.d.s as a function of incident fluence for the two pulse durations. (*d*, *e*) Illustration of how heme atoms are displaced at different snapshots across the simulation replicas. The iron ion in the center is colored green. The value of 8.9 × 10^3^ J cm^−2^ corresponds to 3.0 × 10^18^ W cm^−2^ for a 3 fs pulse and 8.9 × 10^17^ W cm^−2^ for a 10 fs pulse.

We assess structural damage via the root-mean-square displacement (r.m.s.d.) of the heme group based on trajectory files from radiation-damage-resolved molecular dynamics. For each pulse duration and selected fluence values, we averaged the results over 20 simulation replicas. The resulting r.m.s.d.s for the two pulse durations are presented in Figs. 5 ▸(*a*) and 5 ▸(*b*) and show that ion motion increases near the peak of the pulse and becomes fluence-dependent. From these results, we compute the pulse-weighted r.m.s.d. shown in Fig. 5 ▸(*c*). We do not simulate the exact experimental intensities (∼3 × 10^17^ W cm^−2^). Instead, we intentionally explore fluence values that are higher than the experimental conditions to establish an upper bound on possible radiation damage and to guide future experiments based on current and potential future XFEL facility capabilities. Even under these higher fluence conditions, the r.m.s.d.s remain below 0.2 Å, which is lower than the resolution of the reconstructed structure. For the 10 fs pulse, we find that the displacement of the atoms is <0.5 Å for intensities below 1 × 10^19^ cm^−2^, while for the 3 fs pluse the displacement remains <0.2 Å. Because no significant displacement occurs at fluence values even higher than experimental conditions, the results support the conclusion that no detectable radiation damage occurs. We also examined the r.m.s.ds of the iron ion separately. These are presented in Supplementary Fig. S12, where we find similar observations.

We also compare the displacement of atoms in the heme in Figs. 5 ▸(*d*) and 5 ▸(*e*). Here we select a fluence of 8.9 × 10^3^ J cm^−2^, roughly three times higher than that used in the experiment, where we begin to see significant motion in the heme atoms (additional results at 5 × 10^5^ J cm^−2^ are shown in Supplementary Fig. S13). Due to its heavier mass, the iron ion (shown in green) moves less than the lighter atoms in all cases. This has the effect that the Bragg signal originating from the lighter atoms will terminate earlier than the signal from iron, causing the scattering from the Fe atoms to continue even after the contributions from the rest of the protein structure to the Bragg signal have ended. This does not necessarily mean that the Fe atoms will appear stronger in the reconstructed density since the accumulated Bragg signal will no longer be interpretable in terms of the Fourier transform of a single quantity.

## 5. Discussion and conclusions

In this study, we have compared the experimental data from serial femtosecond crystallography (SFX) conducted at the X-ray free-electron laser LCLS in Stanford. The data were collected using 3 and 10 fs X-ray pulses at similar peak pulse intensities. To increase the sensitivity of our experiment to radiation effects, and to examine the effects of high photon absorption on the metal ion, we tuned the photon energy to 7.15 keV, just above the iron absorption edge. The quality of the 10 fs data is comparable to that of the 3 fs data, indicating that the data collected are equally affected by radiation damage. Moreover, the correlation between the intensities of the structure factors, measured with the two different pulse lengths, is very high and exceeds the correlation between the two halves of the individual datasets. The molecular structure achieved from the experimental data is also similar for the two pulse durations. Since 10 fs pulses at the same intensity are a continuation of the exposure experience with the 3 fs pulses, the lack of any discernible radiation effects implies that changes would only be seen at this intensity with longer pulse durations. The resulting *R*_work_/*R*_free_ for both reconstructed structures are almost equal, signaling the fact that there is no observable indication of higher global radiation damage in the case of the 10 fs pulse. Reconstructed electron density around Fe atoms has only a small observable difference for the two pulse durations.

The pulse duration and the pulse energies presented in the work were not explicitly measured. Instead, we rely on information provided by the beamline diagnostics. The pulse energy varies from shot to shot, and we have a variation in the overlap between the X-rays and the free-flowing crystals of different sizes. This generates a wide spread of exposure on the samples, shown in the peakograms in Supplementary Fig. S3. The normalized average photon counts in the Bragg peaks, together with the pulse-energy diagnostics from the beamline, informed us about the approximate photon intensity to which the individual samples were exposed.

We conducted collisional–radiative and molecular-dynamics simulations to study the ionization and dynamics of the crystal. The simulation corroborated the experimental observations of no significant electronic or structural damage at the intensities achieved in the experiment. To access higher Bragg photon counts for diffraction with nanosized crystals, we explored fluence values beyond the experimental conditions. The simulations predict that electronic damage occurs first, altering the atomic scattering factors of atoms and thereby affecting the reconstructed electron density, potentially leading to an incoherent contribution to the Bragg signal that hinders accurate structural refinement. Later in the X-ray exposure, the atoms displace, giving rise to Bragg termination and a possible loss of spatial resolution of the reconstructed electron density. Given that the loss of spatial coherence leads to a loss in Bragg diffraction signal, ion motion could be less detrimental to the resolution. Correlated motion preserved in heavier atoms could affect these conclusions.

In summary, we observe no atomic displacement nor a decrease in scattered signal under either of the two experimental conditions. The 10 fs pulses give equally good structural data as the 3 fs pulses. We see that there is no advantage in decreasing the pulse duration in protein crystallography measurements without increasing the intensity to compensate for the lower fluence.

## Supplementary Material

PDB reference: 3 fs data set, 9tli

PDB reference: 10 fs data set, 9tlj

Raw data.: https://doi.org/10.60883/b34e06428f

Processed data.: https://syncandshare.desy.de/index.php/s/BYg5RmQKcP9xRoE

Supplementary Figures and Tables. DOI: 10.1107/S2059798326008569/gm5123sup1.pdf

## Figures and Tables

**Table 1:** Data-processing and structure-refinement statistics

Pulse duration	3 fs	Attenuated 10 fs
Pulse peak fluence (J cm^−2^)	8.0 × 10^2^	3.4 × 10^3^
Pulse peak intensity (W cm^−2^)	2.7 × 10^17^	3.4 × 10^17^
Resolution range (Å)	29.16–2.46 (2.55–2.46)	29.16–2.46 (2.55–2.46)
Space group	*P*2_1_2_1_2_1_	*P*2_1_2_1_2_1_
*a*, *b*, *c* (Å)	62.51, 80.97, 111.48	62.51, 80.97, 111.48
α, β, γ (°)	90, 90, 90	90, 90, 90
No. of crystals/No. indexed	130296/129124	111905/120329
CC*	0.998 (0.595)	0.999 (0.745)
CC_ano_	0.141	0.187
*R*_split_ (%)	7.33 (125.6)	5.31 (110.0)
〈*I*/σ(*I*)〉	17.13 (1.51)	21.08 (3.17)
Completeness (%)	97.01 (38.6)	97.62 (50.82)
Multiplicity	466.37 (5.9)	435.00 (5.3)
Unique reflections	21158 (2067)	21158 (2067)
Wilson *B* factor (Å^2^)	31.38	33.88
*R*_work_ (%)	17.77 (24.85)	17.86 (26.80)
*R*_free_ (%)	23.48 (31.49)	23.01 (34.42)
No. of solvent molecules	86	102
No. of protein residues	572	573
R.m.s.d., bond lengths (Å)	0.002	0.003
R.m.s.d., angles (°)	0.56	0.64
Clashscore	3.31	3.39
Average *B* factor (Å^2^)	41.93	40.61

## Data Availability

The PDB entries of the deposited structures are 9tli for the 3 fs data set and 9tlj for the 10 fs data set. Processed data (in the form of *CrystFEL*.stream files) can be downloaded from https://syncandshare.desy.de/index.php/s/BYg5RmQKcP9xRoE. The raw data can be found at https://doi.org/10.60883/b34e06428f. If you have issues trying to download the raw data please contact the authors.
