# Supplementary material for: Radiation damage in serial femtosecond crystallography studied in hemoglobin
Source: Acta Crystallogr D Struct Biol. 2026 Sep 30;82(Pt 10):1169–80. doi: 10.1107/S2059798326008569 (PMC13632746; doi:10.1107/S2059798326008569)
Supplement: Supplementary file 1 [file d-82-01169-sup1.pdf]

STRUCTURAL BIOLOGY

Volume 82 (2026)

Supporting information for article:

Radiation damage in serial femtosecond crystallography studied in hemoglobin

Marina Galchenkova, Ibrahim Dawod, Sebastian Cardoch, Janina Sprenger, Dominik Oberthür, Salah Awel, Emiliano De Santis, Oscar Grånäs, Juraj Knoska, Markus Metz, Spencer Passmore, Gisel Pena, Iosifina Sarrou, Max Wiedorn, Nicusor Timneanu, Henry Chapman, Oleksandr Yefanov and Carl Caleman

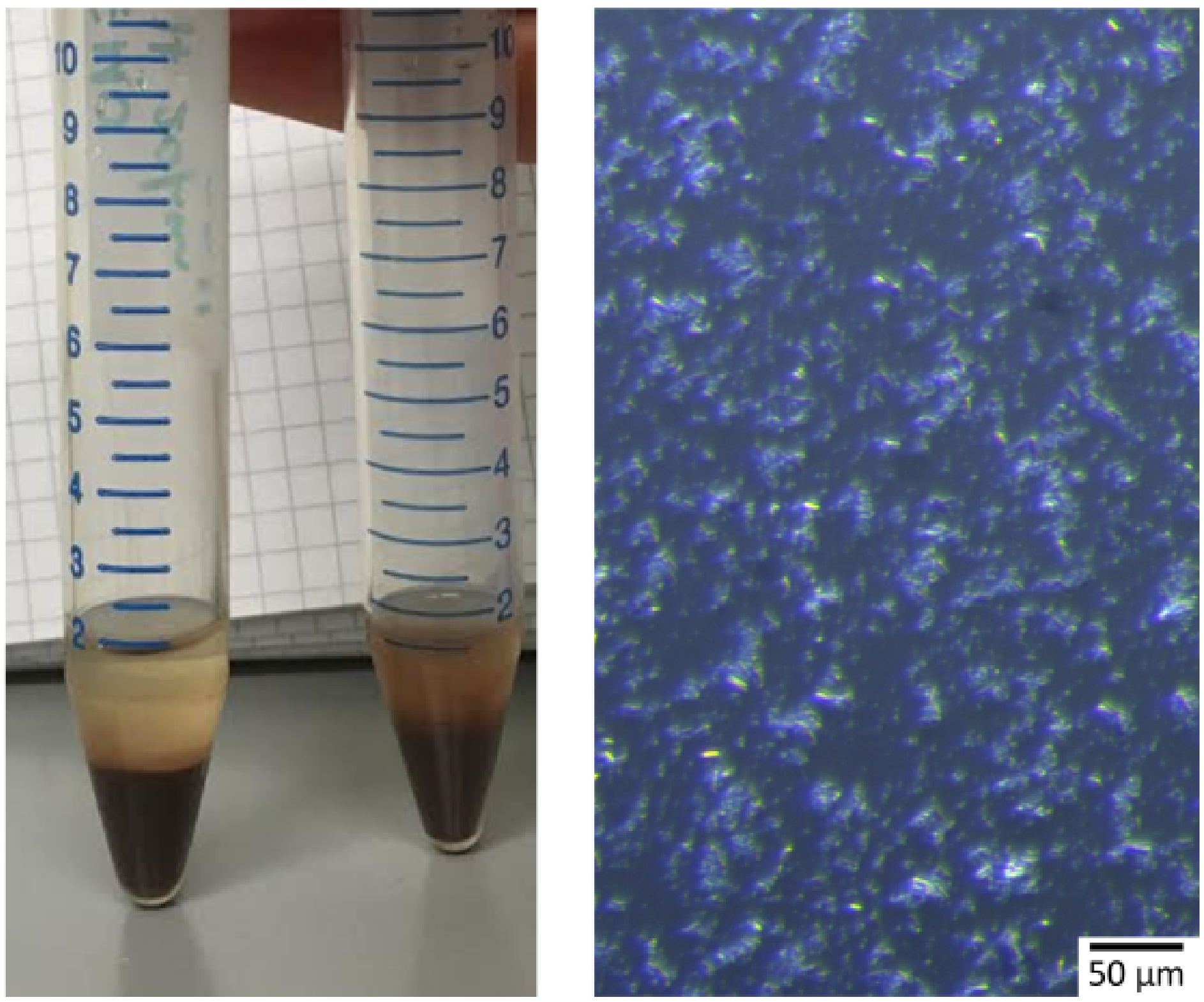

Figure S1: The photographs displayed in this section are sourced from (Knoška *et al.*, 2020) and visually represent the hemoglobin crystallization process. The left image depicts the outcome of the process, showing the hemoglobin sample after successful crystallization. In this image, the crystals are settled at the bottom of the falcon tubes, indicating the successful formation of well-defined crystals. The right image presents a micrograph of the crystalline slurry, revealing the presence of crystals with sizes as small as one micrometre. This micrograph provides a closer look at the individual crystals within the slurry, highlighting their size and structure. These photographs visually depict the hemoglobin crystallization process and the resulting crystal samples.

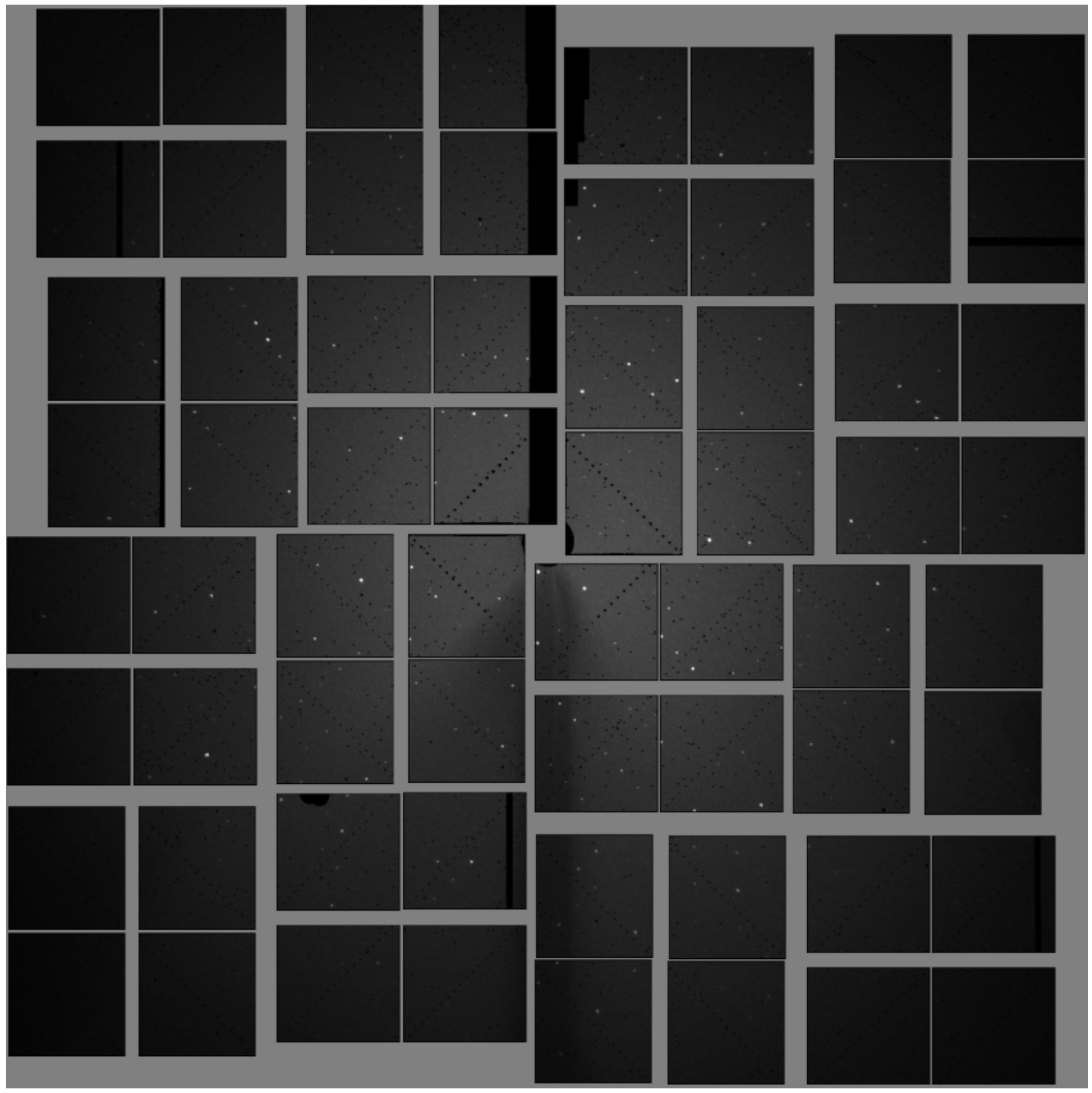

Figure S2: A diffraction pattern obtained from the LR17 beamtime using 2.3M CS-PAD detector. The pattern contains a series of white spots, representing the constructive interference of X-rays scattered by the crystal's atomic structure. The visible black dots are the unbonded pixels.

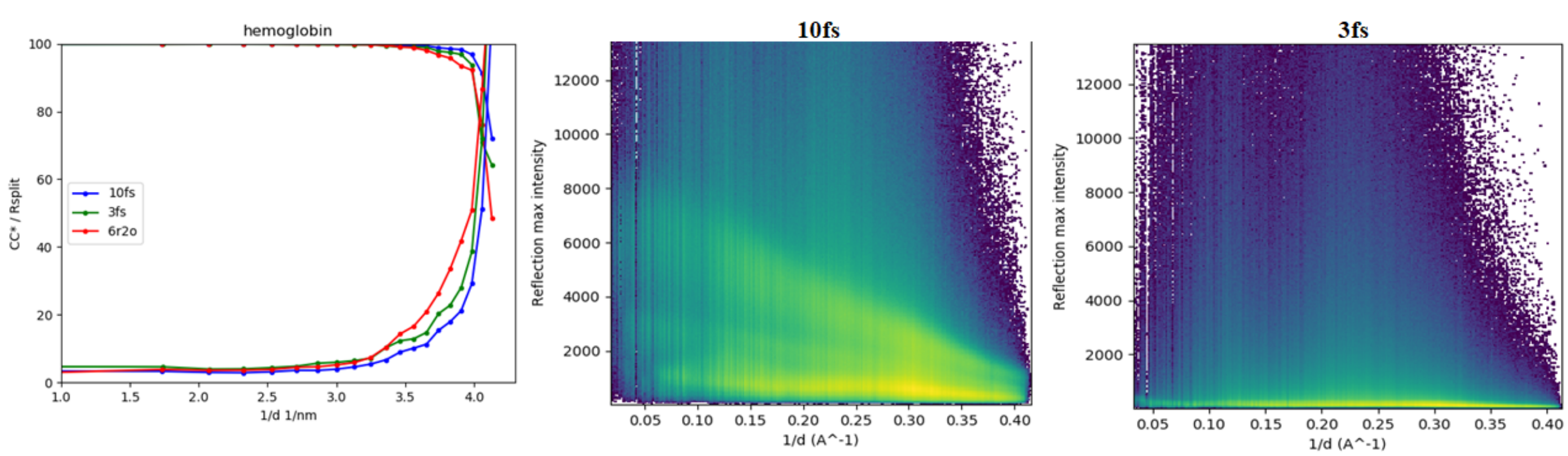

Figure S3: Data statistics for the experimental data. Left plot shows a comparison between the CC^*/R_{split} of the reprocessed data using pulse duration of 3 fs and 10 fs, along with the published data corresponding to the PDB ID 6R2O. The middle and the right figures present peakograms for the complete datasets measured with 10 fs and 3 fs pulse durations, visually representing the distribution of the highest intensities of the diffraction peaks. The diffraction peaks containing saturated pixels (with values above 13500) were excluded from processing.

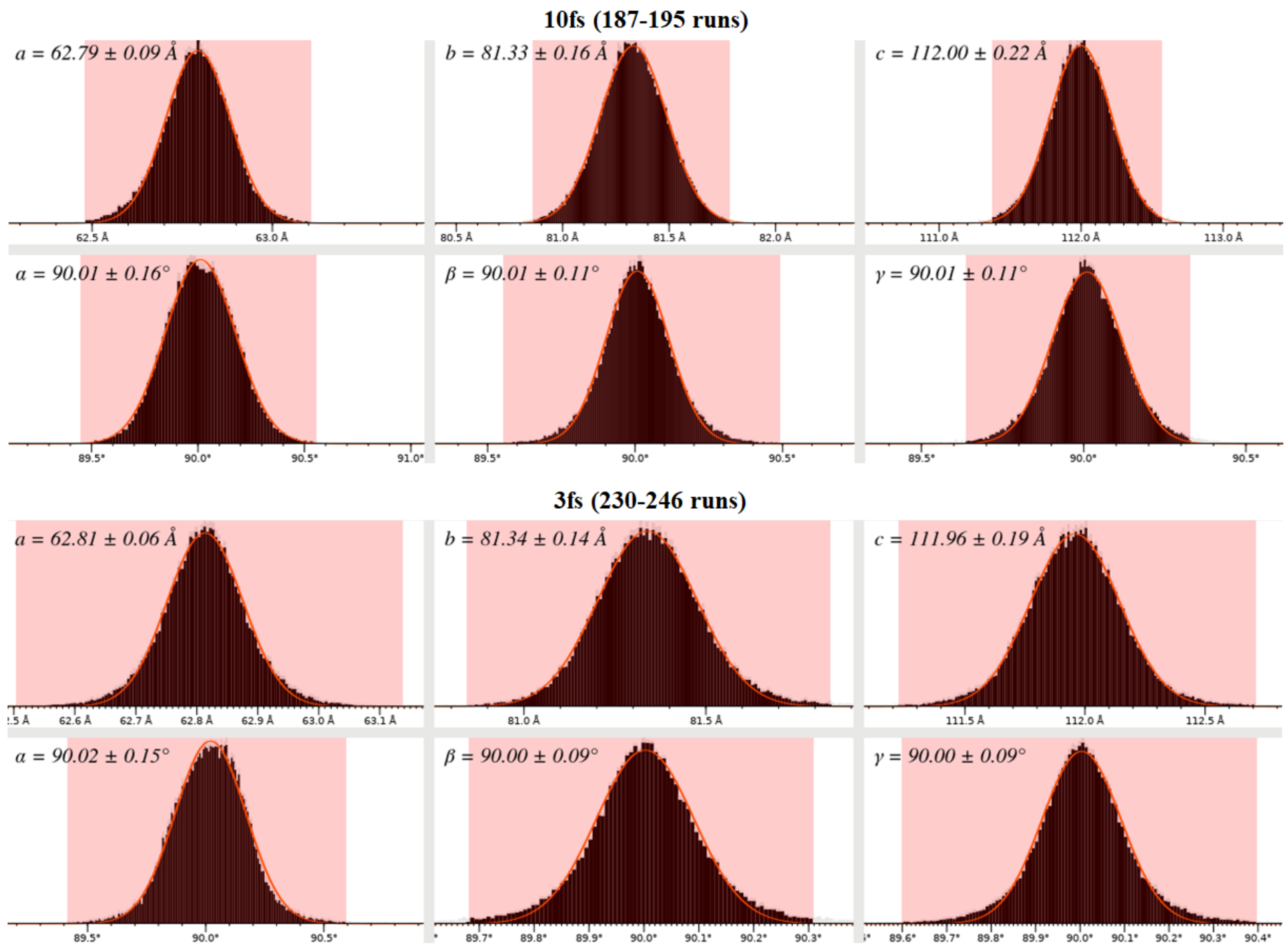

Figure S4: The distribution of unit cell parameters for subsets of the hemoglobin datasets collected with pulse duration of 10 fs and 3 fs. The unit cell parameters provide essential information about the size and symmetry of the crystals.

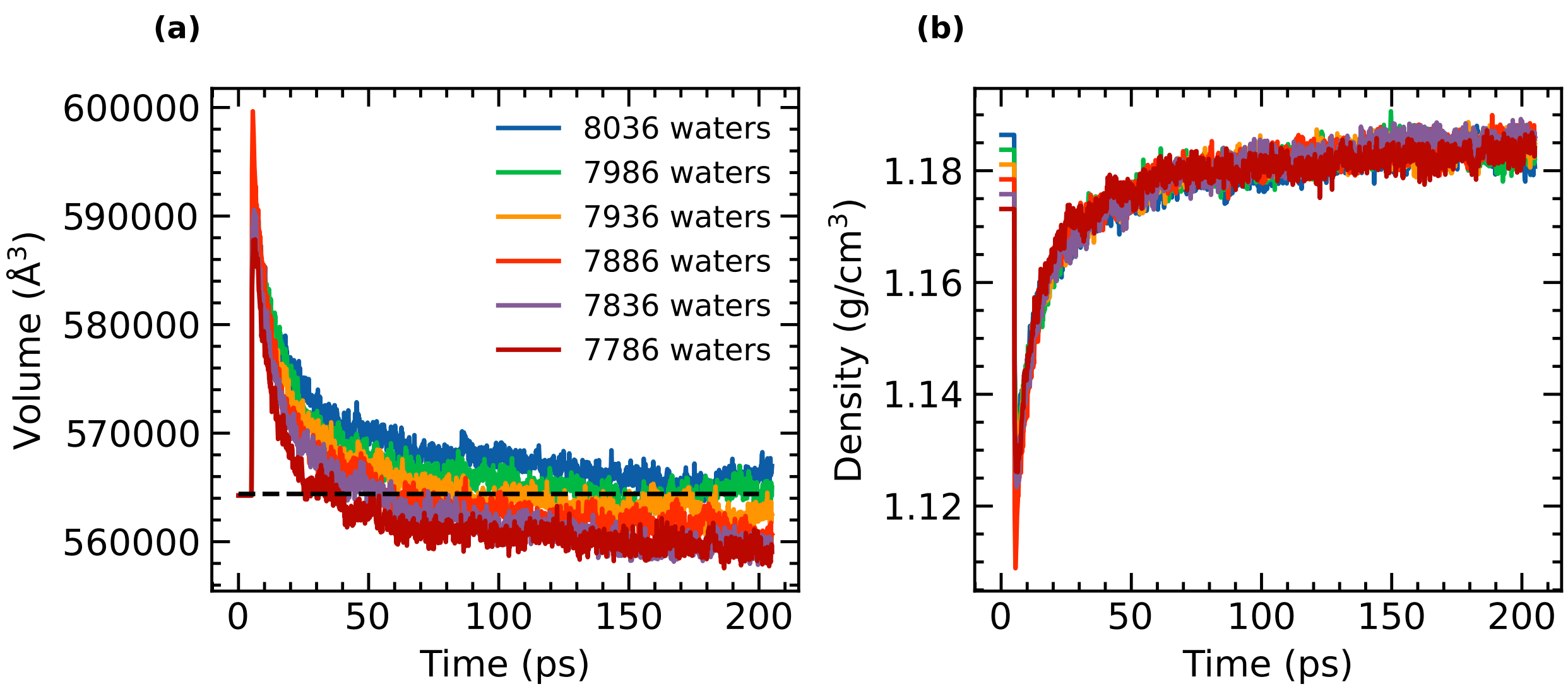

Figure S5: Determining the simulated crystal's (a) solvation and (b) density based on changes to its volume. We find that approximately 7986 water molecules are needed to preserve the starting volume of the system (dashed line) determined from experimental data.

Table S1: Extended table of data processing and structure refinement statistics.

**Pulse duration**	3 fs	attenuated 10 fs
**Resolution range (Å)**	29.16 - 2.46 (2.55 - 2.46)	29.16 - 2.46 (2.55 - 2.46)
**Space group**	P 21 21 21	P 21 21 21
**Unit cell (Å),(°)**	62.51 80.97 111.48, 90 90 90	62.51 80.97 111.48, 90 90 90
**N. indexed patterns/crystals**	130296 / 129124	111905 / 120329
**$CC_{1/2}$**	0.992 (0.215)	0.996 (0.385)
**CC^***	0.998 (0.595)	0.999 (0.745)
**CC_{ano}**	0.141	0.187
**R_{split} (%)**	7.33 (125.6)	5.31 (110)
**$l/\sigma(l)$**	17.13 (1.51)	21.08 (3.17)
**Completeness (%)**	97.01 (38.6)	97.62 (50.82)
**Multiplicity**	466.37 (5.9)	435.00 (5.3)
**Unique reflections**	21158 (2067)	21158 (2067)
**Wilson B-factor ($Å^2$)**	31.38	33.88
**R_{work} (%)**	17.77 (24.85)	17.86 (26.80)
**R_{free} (%)**	23.48 (31.49)	23.01 (34.42)
**Number of non-hydrogen atoms**	4804	4848
**Number of macromolecules**	4536	4564
**ligands**	182	182
**solvent**	86	102
**Protein residues**	572	573
**RMS-bonds (Å)**	0.002	0.003
**RMS-angles (°)**	0.56	0.64
**Ramachandran favored (%)**	98.23	98.58
**Ramachandran allowed (%)**	1.77	1.42
**Ramachandran outliers (%)**	0.00	0.00
**Rotamer outliers (%)**	1.66	1.24
**Clashscore**	3.31	3.39
**Average B-factor ($Å^2$)**	41.93	40.61
**macromolecules**	41.89	40.53
**ligands**	40.95	39.00
**solvent**	46.09	47.07

Table S2: Values for Anomalous peaks aligned with the four Fe atoms of the 10 fs and 3 fs datasets, in values of σ.

**10 fs**	**3 fs**
23.79	19.66
22.26	18.05
19.83	14.61
13.69	12.07

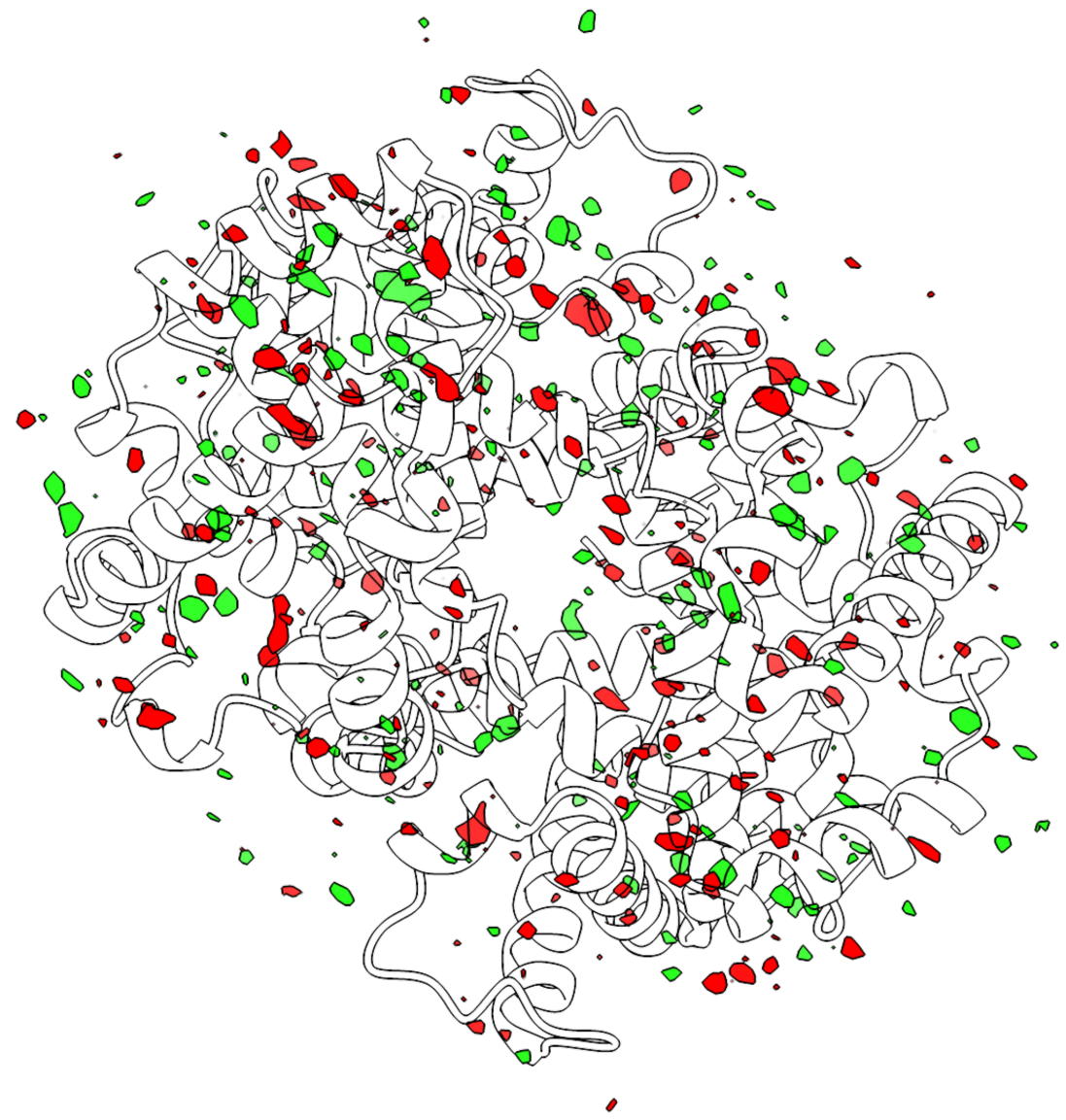

Figure S6: Fo-Fo-map scaled at $\pm 3\ \sigma$, the unbiased model for the 3 fs dataset coming out of the CRANK2-pipeline was used for phasing. On the right hand side final heme-positions (hemes shown in blue) are plotted for orientation. Created in https://BioRender.com.

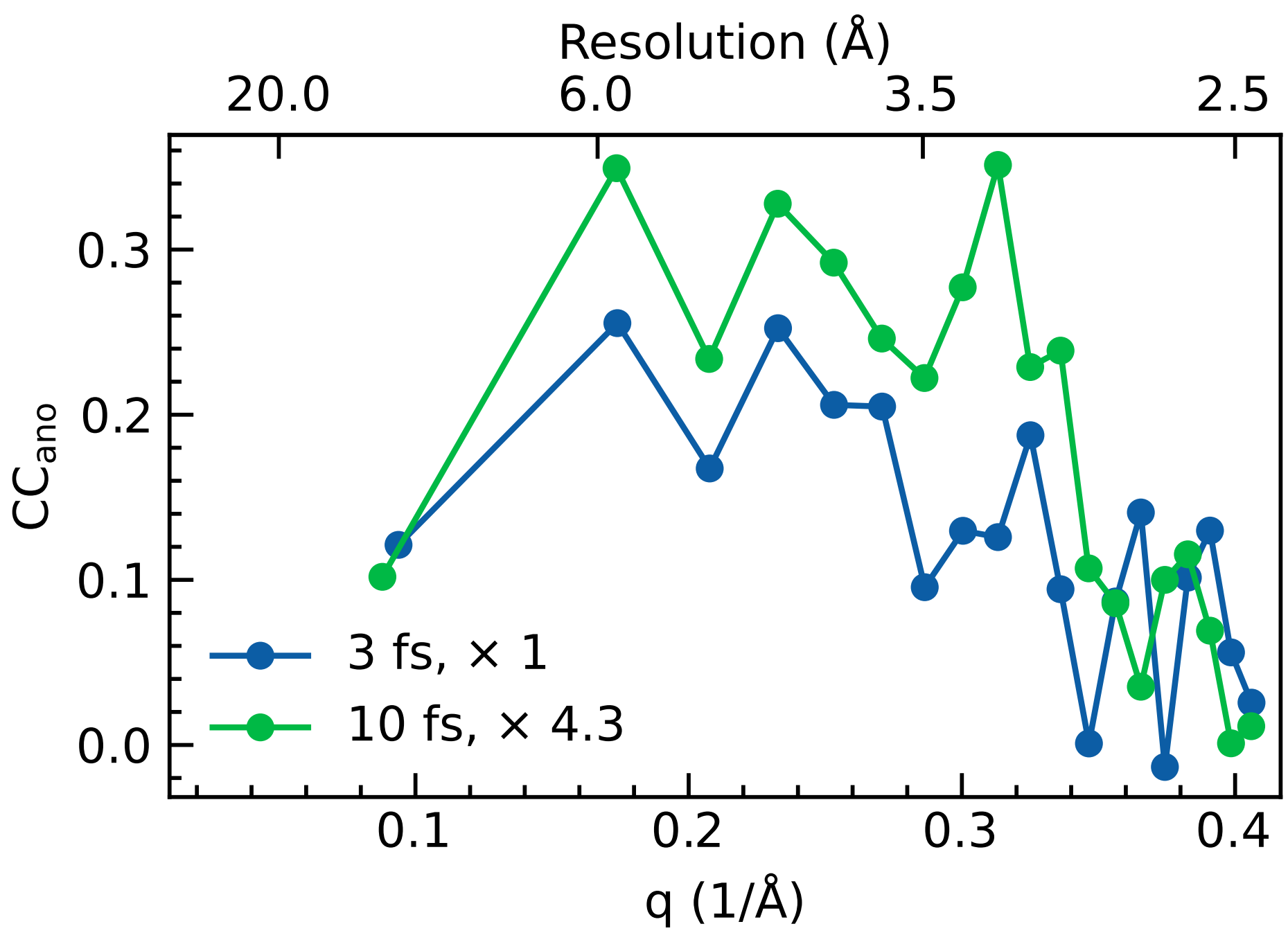

Figure S7: The anomalous statistics plot (CC_{ano}) for the two datasets measured with 3 fs and 10 fs pulses.

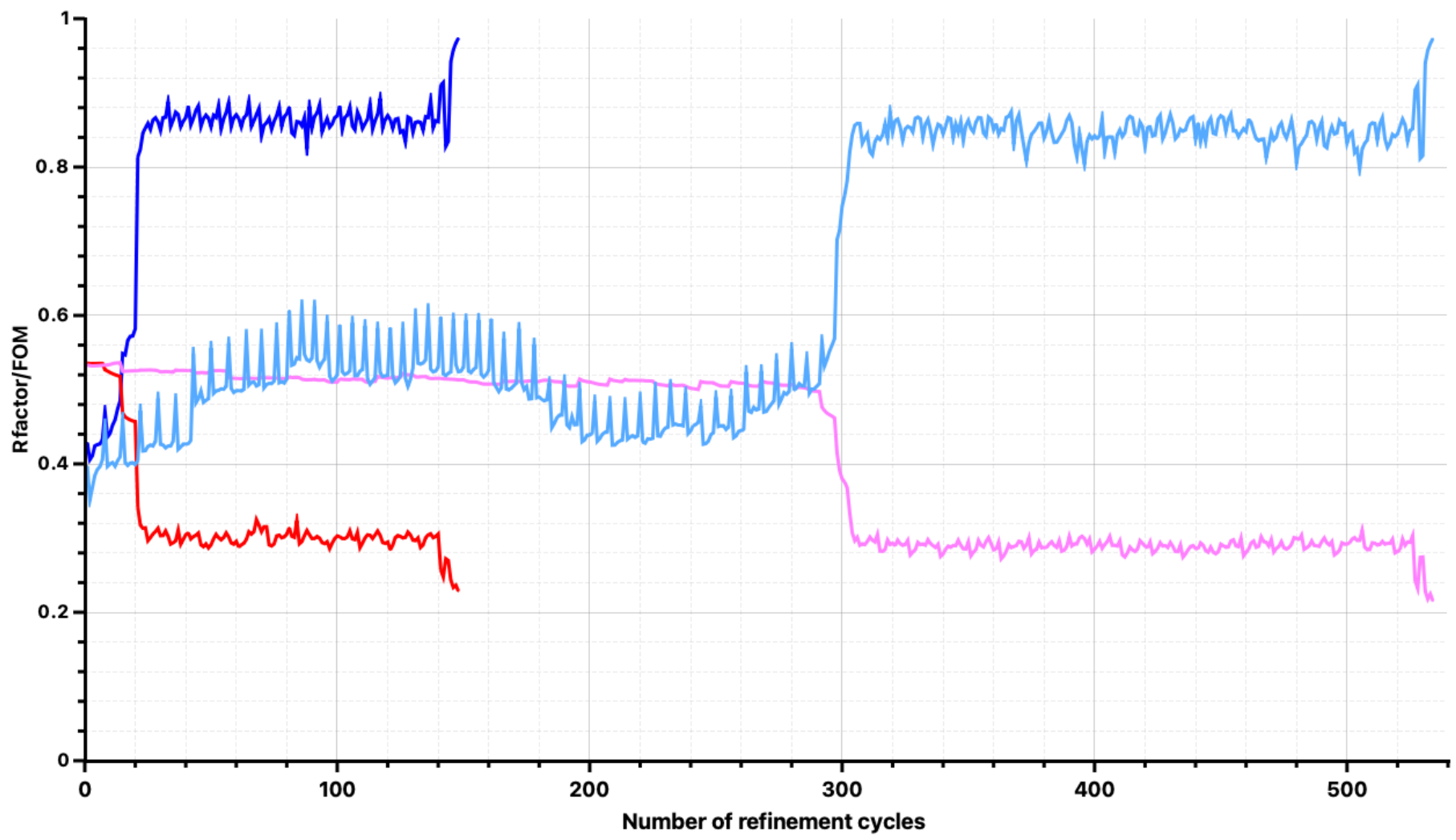

Figure S8: Evolution of refinement quality metrics during iterative CRANK2 refinement/automatic model building cycles (with Refamc and Buccaneer) after phasing with SHELXC/D/E for the 10 fs and 3 fs datasets. The R-factor values are shown in red (10 fs) and magenta (3 fs), while the corresponding figures of merit (FOM) are shown in blue (10 fs) and light blue (3 fs). Both datasets exhibit distinct convergence behavior during refinement, with the 10 fs dataset converging substantially faster and reaching a final FOM/R-factor of 0.9737/0.2285. The 3 fs dataset converges more slowly but attains a slightly lower final R-factor, with a final FOM/R-factor of 0.9731/0.2153.

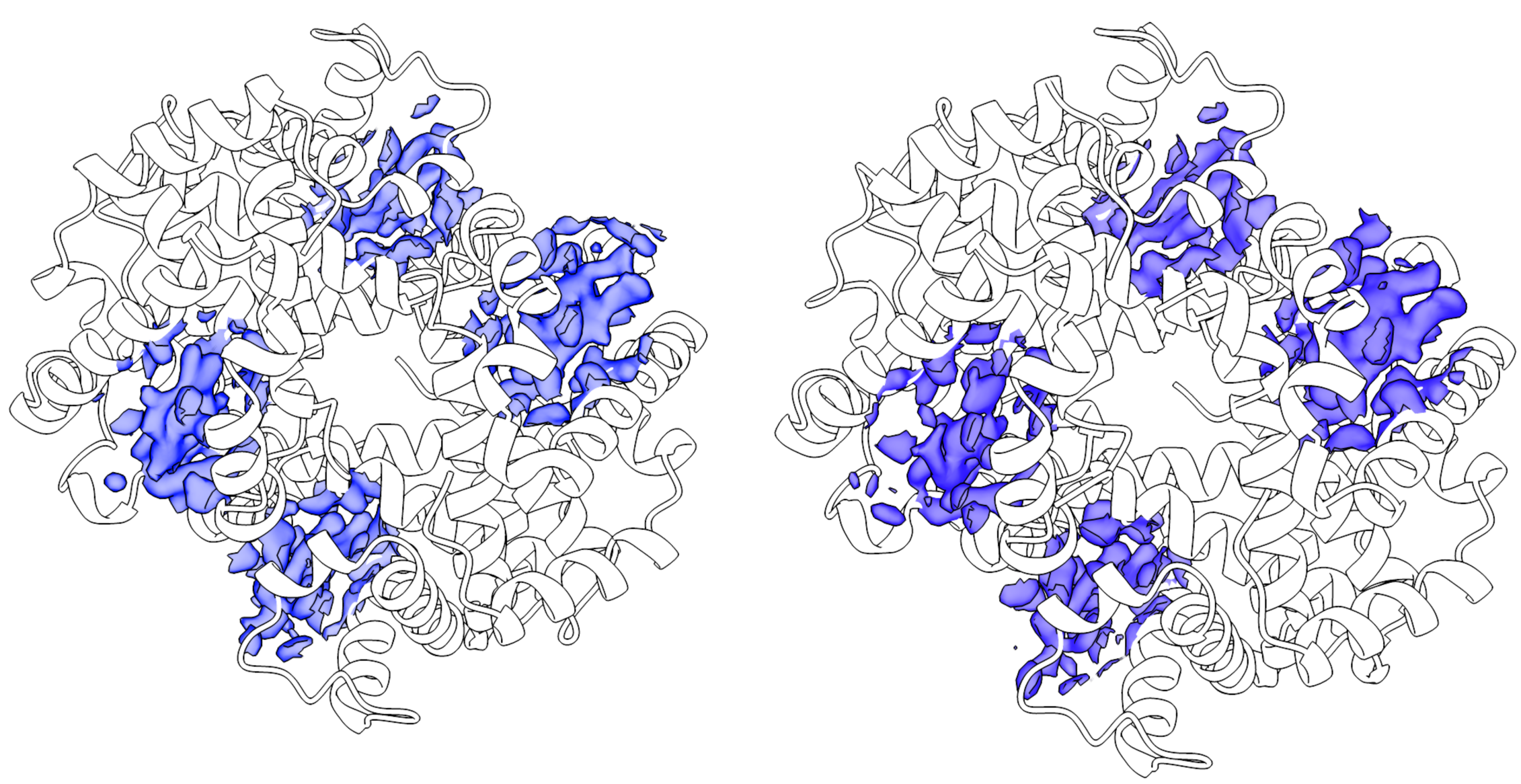

Figure S9: 2mFo-DFc map in blue (output from CRANK2-pipeline) around heme-positions (no heme in model) scaled at 1 σ and plotted on top of automatically generated model from CRANK2-pipeline, based on experimental phasing with SHELXC/D/E. Left hand side: 3 fs dataset, right hand side: 10 fs dataset. Created in https://BioRender.com.

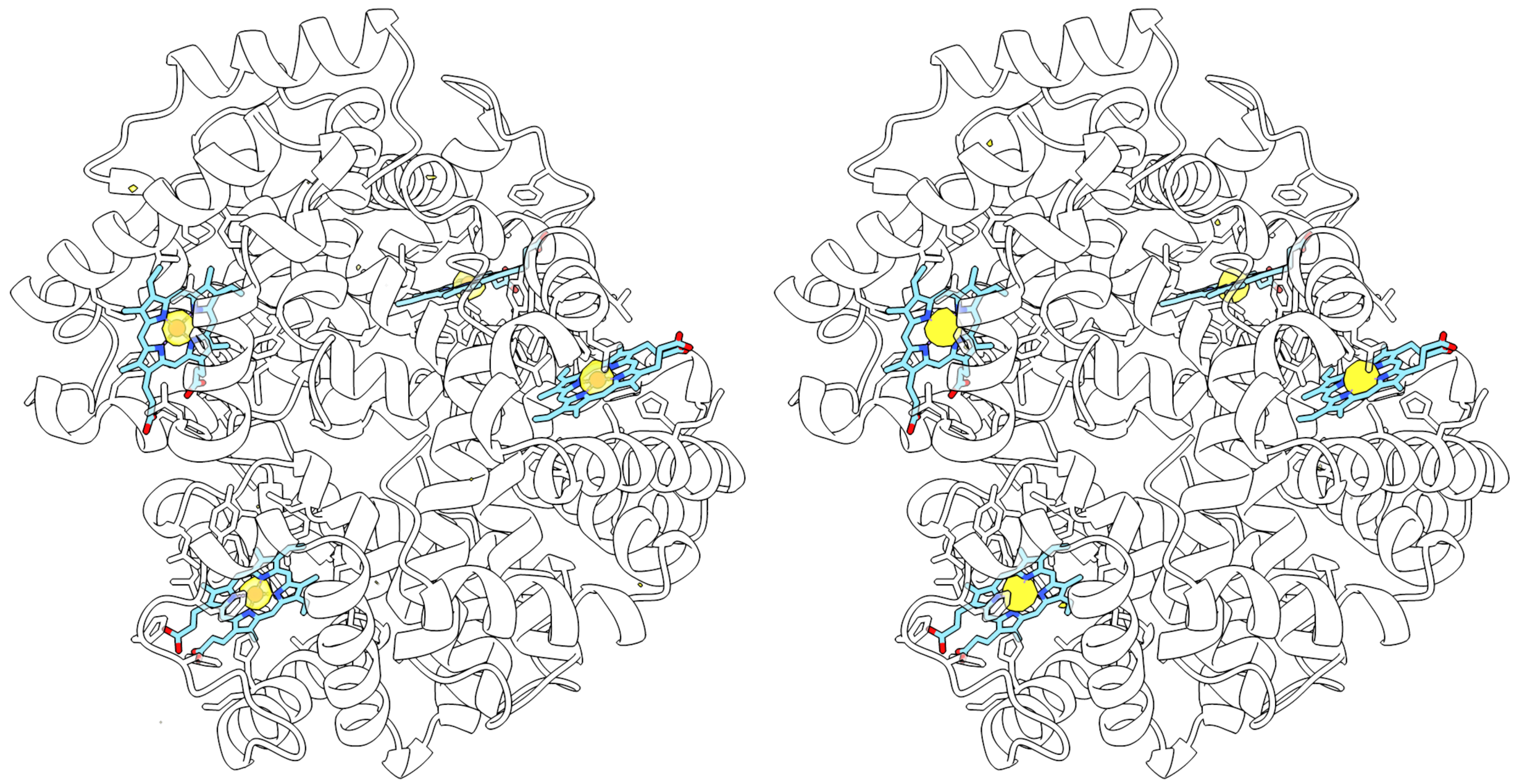

Figure S10: Anomalous maps scaled at 4 σ plotted with refined structures, left hand side: 3 fs dataset, right hand side: 10 fs dataset. Iron ions in yellow, and heme group in blue. Created in https://BioRender.com.

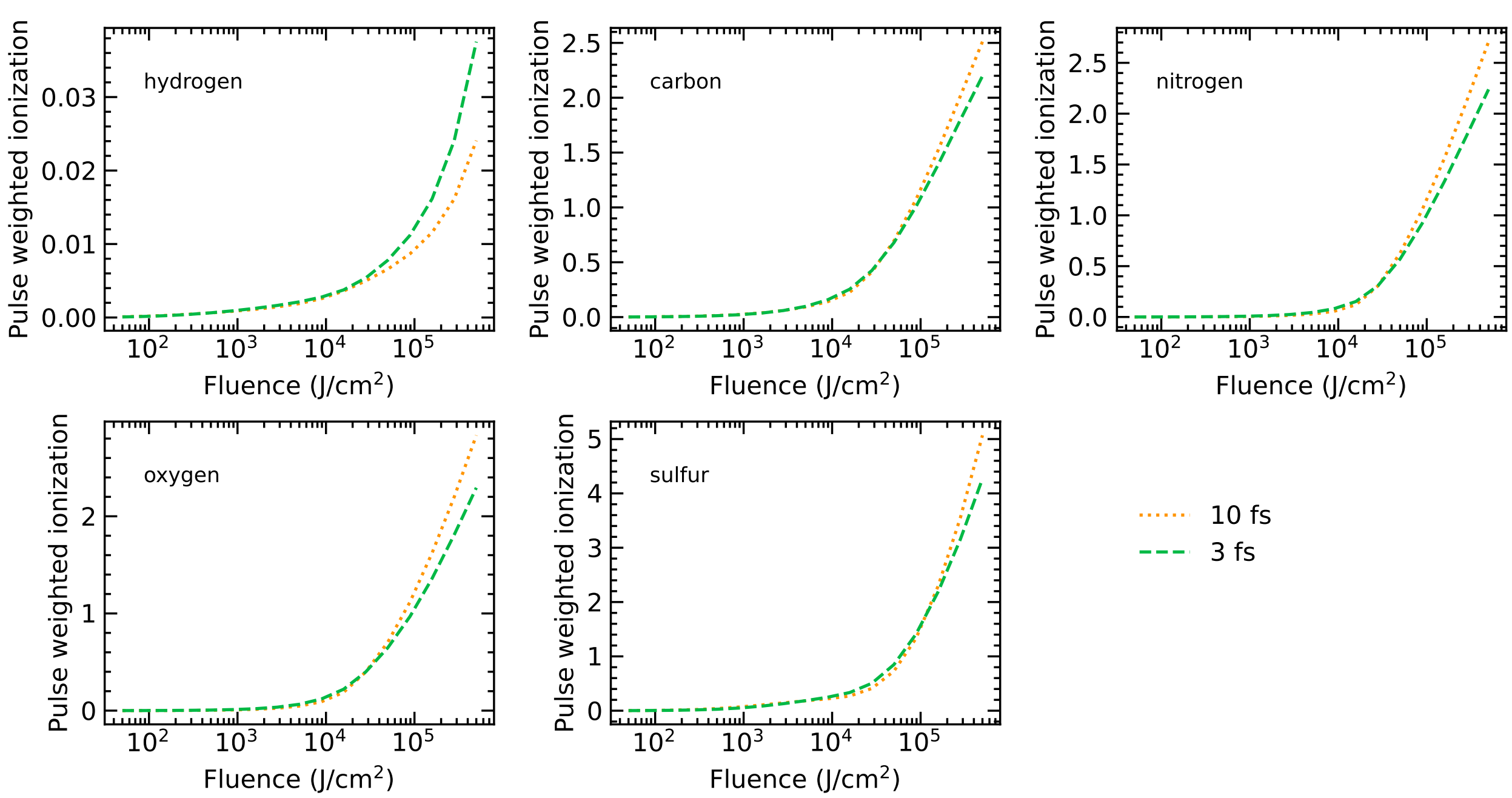

Figure S11: Pulse-weighted ionization predicted by collisional radiative simulations as a function of incident X-ray pulse fluence for atomic species found in hemoglobin for 10 fs and 3 fs.

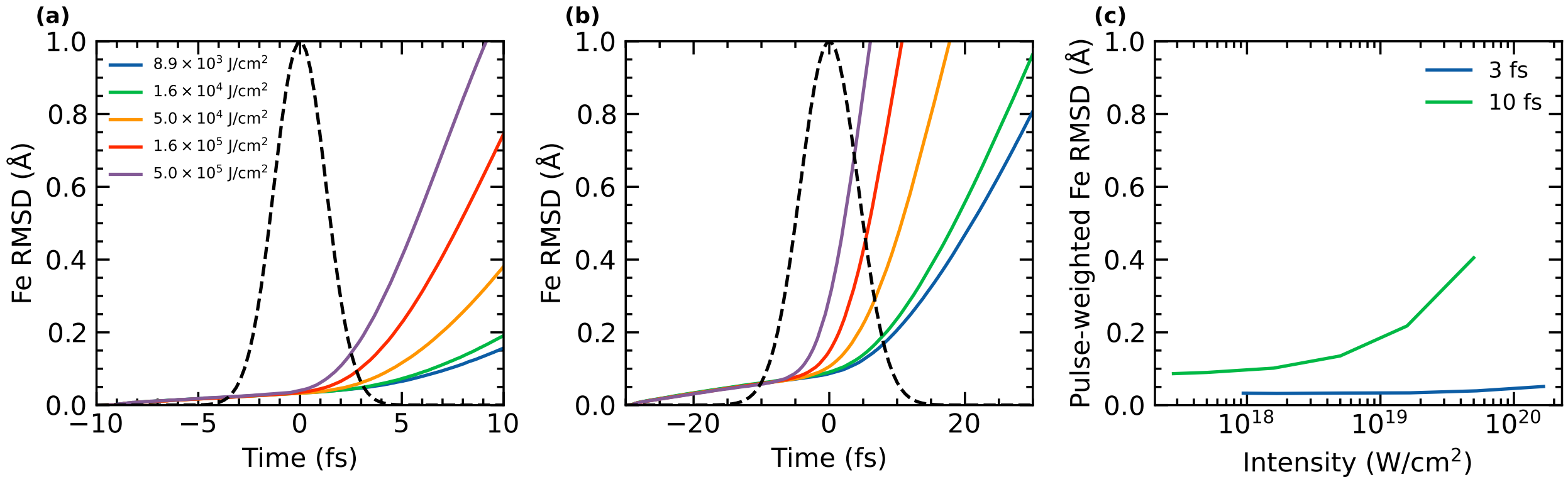

Figure S12: Root-mean-square displacement (RMSD) for the iron atoms found in the heme group at different fluence values for (a) 3 fs and (b) 10 fs pulse durations. (c) Average Fe RMSD weighted by the pulse shape.

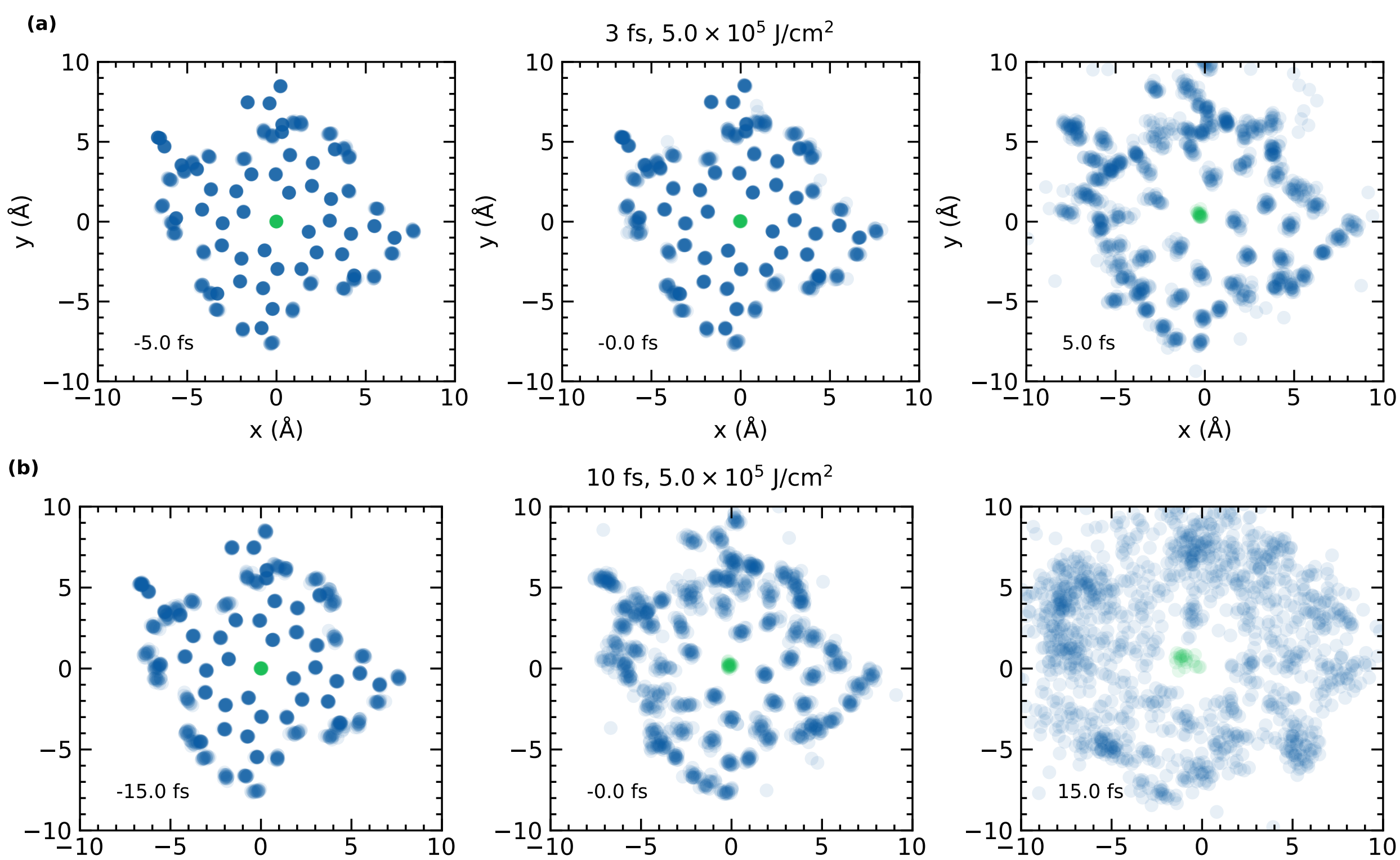

Figure S13: Average displacement of a selected heme group over the simulation replicas for the 3 fs and 10 fs pulse durations with an incident fluence of 5×10^5 J cm^{-2}.
